# Stress symptoms and associated factors among adolescents in Dhaka, Bangladesh: findings from a cross-sectional study

**DOI:** 10.1186/s12888-022-04340-0

**Published:** 2022-12-19

**Authors:** Afifa Anjum, Sahadat Hossain, M. Tasdik Hasan, Enryka Christopher, Md. Elias Uddin, Md. Tajuddin Sikder

**Affiliations:** 1 Department of Public Health and Informatics, Jahangirnagar University, Savar, Dhaka, 1342 Bangladesh; 2 Department of Public Health and Informatics, Bangabandhu Sheikh Mujib Medical University, Shahbag, Dhaka, 1000 Bangladesh; 3 Department of Behavioural Science and Health, Institute of Epidemiology and Health Care, University College London (UCL), London, England; 4 Action Lab, Department of Human Centred Computing, Faculty of Information Technology, Monash University, Melbourne, Australia; 5 Department of Public Health, State University of Bangladesh, Dhaka, Bangladesh; 6 Department of Primary Care and Mental Health, University of Liverpool, Liverpool, UK; 7 Trauma and Community Resilience Center, Boston Children’s Hospital & Harvard Medical School, Boston, USA; 8 Department of English, University of Dhaka, Dhaka, 1000 Bangladesh

**Keywords:** Stress, Physical activity, Lifestyle, Screens, Social media, Sleep, Adolescents, Bangladesh

## Abstract

**Background:**

Stress affects adolescents’ daily lives by disrupting their working capacity and begets comorbidity. This study aimed to estimate the prevalence of stress symptoms and the factors associated with these symptoms among secondary school-going adolescents in Bangladesh.

**Methods:**

A cross-sectional study using two-stage cluster sampling was conducted. A self-administered questionnaire was given to 2355 adolescents from nine secondary schools in Dhaka, Bangladesh. Of the respondents, 2313 completed the 10-item Perceived Stress Scale (PSS-10). Sociodemographic information, self-reported body image, a modified Leisure Time Exercise Questionnaire (LTEQ), and the WHO Global Physical Activity Questionnaire (GPAQ) were used to determine the sociodemographic and lifestyle factors associated with stress symptoms among adolescents.

**Results:**

Findings suggest that about 65% of adolescents experienced moderate stress symptoms, and about 9% experienced high-stress symptoms. Females (58.7%) suffered more from stress compared to males (41.3%). Age, grade, and residential setting were significantly associated with stress. Logistic regression estimates show that level of physical activity (AOR: 1.52; 95% CI:1.26–1.84), sleep dissatisfaction (AOR: 1.33; 95% CI: 1.07–1.65), and perception of self as overweight/obese (AOR: 1.46; 95% CI: 1.13–1.89) were significantly associated with stress symptoms among adolescents.

**Conclusions:**

Stress symptoms are highly prevalent among secondary school adolescents in Bangladesh. Further exploratory investigations are needed on possible intervention strategies to reduce the burden of stress among adolescents.

**Supplementary Information:**

The online version contains supplementary material available at 10.1186/s12888-022-04340-0.

## Introduction

Adolescence, commonly defined as the period between 10 and 19 years [1], is an important phase of human growth and development, bridging childhood to adulthood. The numerous psychosocial and physiological changes adolescents experience during this time make them especially vulnerable to stress [2]. In view of the magnitude of this transition, it is understandable that this period is often characterized as a tempestuous, stressful stage of life [3].

Identified adolescent stressors include rapid changes in their bodies, sexual awakenings, establishing social networks, and a multitude of others, all of which pose threats to stable development and mental well-being. The most significant mental health issues that adolescent stress contributes to are depression, anxiety, suicide, drug use, and antisocial behaviour [4]. Adolescents with elevated stress levels have been found to indulge in a variety of maladaptive and harmful activities, such as increased alcohol and substance use, unprotected sexual intercourse, physical inactivity, unhealthy eating habits, and poor sleep hygiene [2]. The physiological impact stress has on general wellbeing, as well as specific health outcomes, such as weakened immune systems and diseases (cancer, diabetes, dermatological conditions, etc.), are well known. More recently, cognition, coping mechanisms, and social reinforcement have been identified as mediating and moderating factors between stress and different health consequences [4].

Persistent stress symptoms throughout adolescence can lead to chronic stress, which could have a significant impact on livelihood through factors such as job absenteeism. In Sweden, stress is the most frequently diagnosed reason for long-term sick leave, and adults who experienced chronic stress during childhood suffer most. A 2009Swedish study on children’s living conditions estimated that 60% of female and 38% of male high school adolescents suffer from stress-related problems due to daily life stressors [3]. Deterioration in health, an inevitable result of stress, can take the form of mental fatigue, physical weakness, or cognitive difficulties [5]. Studies have shown that Indian adolescents face additional stressors from cultural influences, such as strong parental expectations, constrictive living arrangements, social hierarchies, and academic concerns, to name just a few [6].

In Bangladesh, a substantial number of studies have been conducted on the mental health of youth or tertiary level students [7, 8]. These studies have measured depression, anxiety, and stress levels. However, most studies research stress along with multiple other symptom measures, and a review of the literature shows a dearth of studies on secondary school-aged youths. Furthermore, not a single study focused exclusively on stress among this important sub-population. But addressing stress among adolescents is necessary since chronic stress has the probability of triggering non-communicable diseases (NCDs), such as high blood pressure, heart disease as well as obesity. Apart from these, persisting stress can lead to other mental health conditions including clinical depression and anxiety, which have become more common among youths [9]. This study aims to fill these gaps in the literature by focusing on factors that influence stress levels in secondary school-aged adolescents. To the best of our knowledge, this is one of the few studies that explored secondary school-aged adolescents’ stress and the factors linked to it in Bangladesh.

## Methods

### Study design and setting

**Fig. 1:**
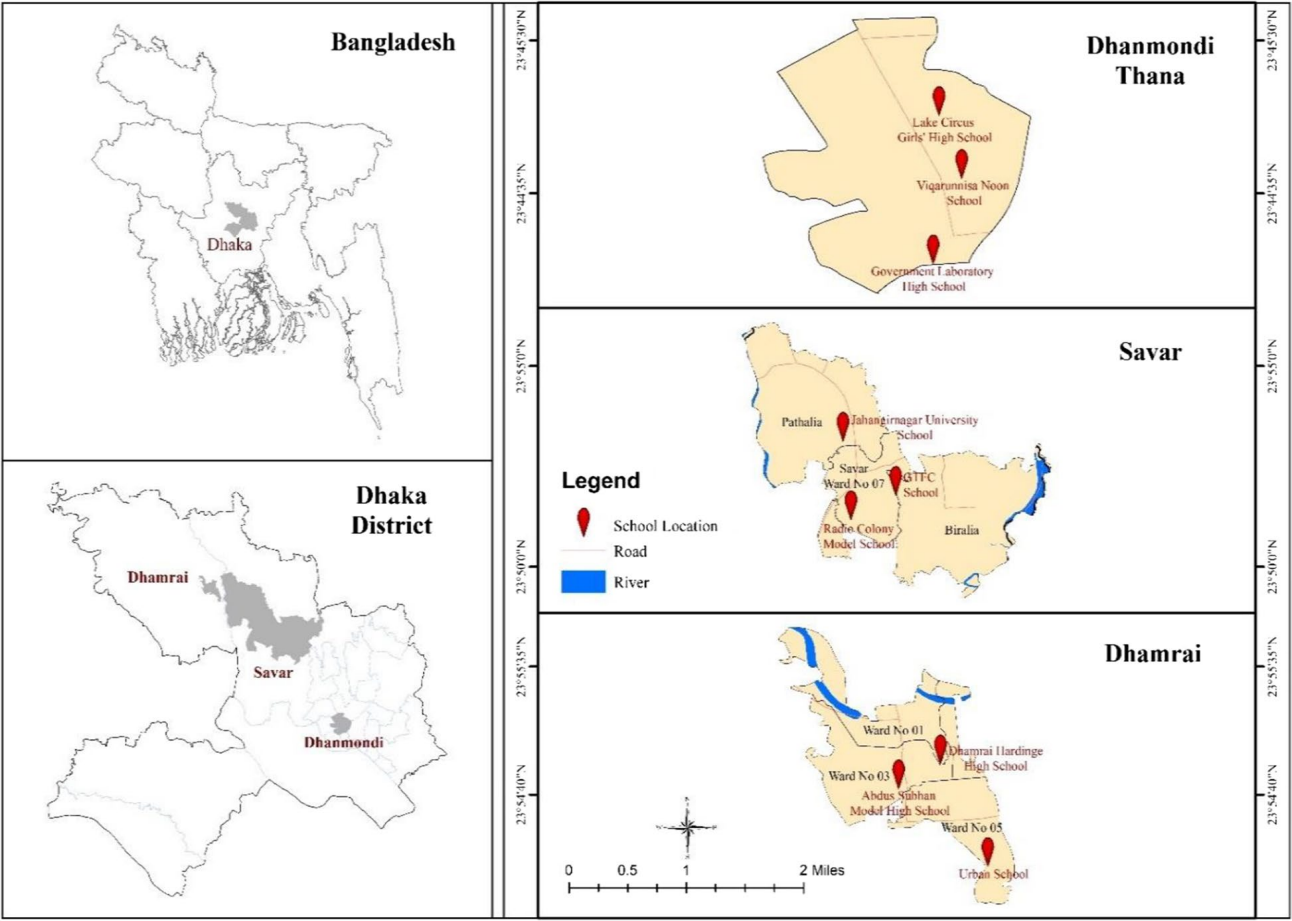
Location map of the study area

This cross-sectional study was conducted between January 2019 and February 2020 among secondary school students in urban, semi-urban, and rural areas of the Dhaka district (Fig. 1). Dhaka is the capital of Bangladesh and, as such, provides robust population variation for sampling. Information on urbanization of each area was based on a combination of factors, including education, health, civil organization, and other factors. A research team with intimate knowledge of the Dhaka region and beyond had several discussions to identify key areas, from which a convenience sample was drawn. Dhanmondi thana within Dhaka city was the urban data collection site. The semi-urban site was Savar thana, located 24 km northwest of Dhaka. The rural site was Dhamrai Upazila, located 40 km northwest of Dhaka. Variability in socio-economic contexts and accessibility of the areas for the research team were considered when choosing these areas. Sampling techniques were used within these areas to select participants.

**Fig. 2:**
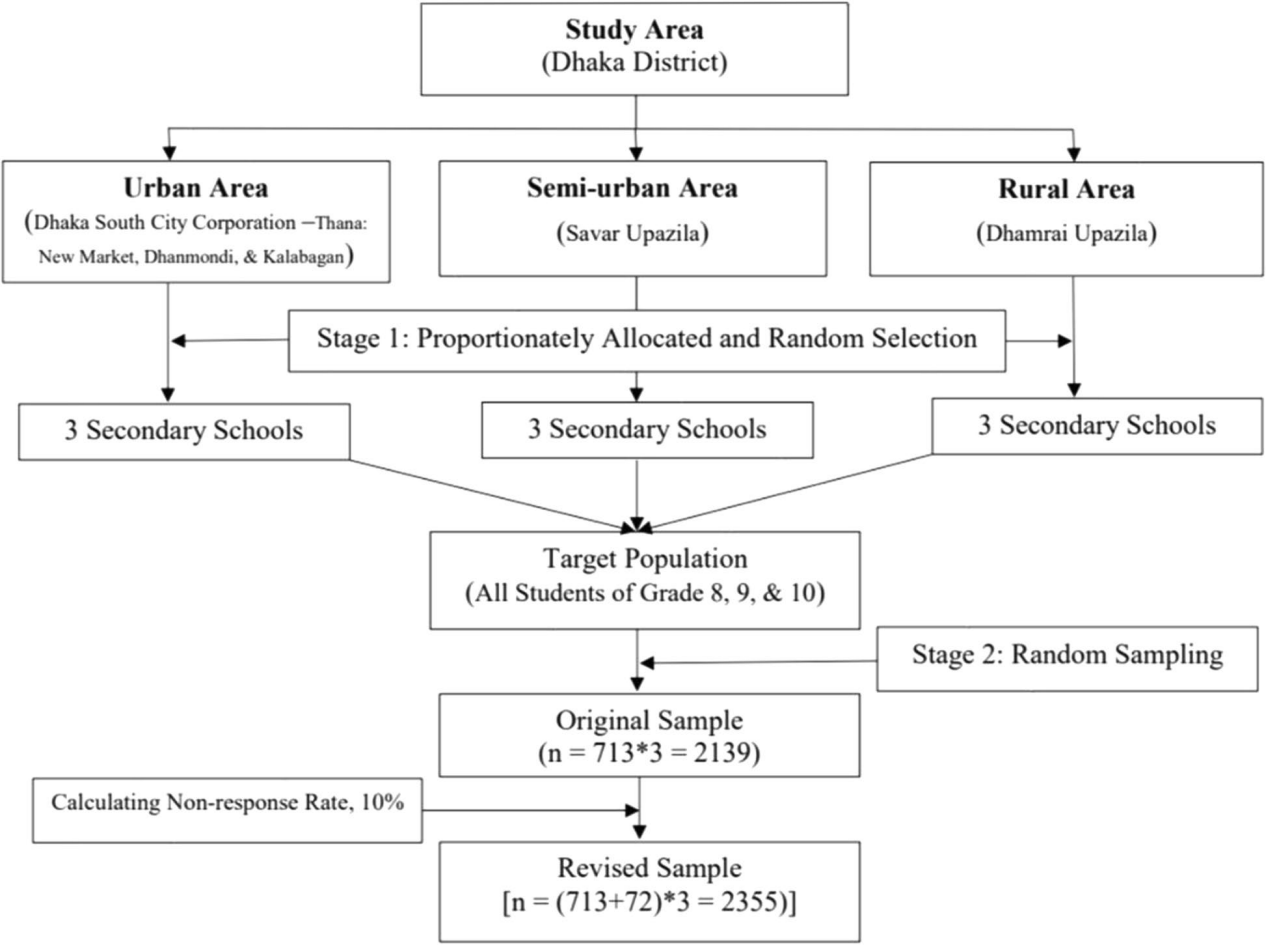
Flow diagram of two-stage sampling procedure

In the first stage of the study, a list of all the secondary schools in the study areas was prepared. Among 16 secondary schools in the Dhanmondi (urban) area, 14 in the Savar (semi-urban) area, and 16 in the Dhamrai (rural) area, three schools were randomly chosen from each collection site (Fig. 2).

### Study population

The targeted population for this study was adolescents of grades 8–10 (12–17 years old), as grades 6–10 are considered secondary education in Bangladesh. A two-stage cluster sampling technique was followed, and the n (713) was multiplied by the three study sites for a minimum sample size of 2139. Using a 10% non-response rate, a target recruitment of 2353 respondents was calculated for this study.

### Data collection procedure

Before field implementation of the study, student lists of grades 8, 9, and 10 were collected from respective school authorities. With the support of the headmasters/principals of the schools and the teachers of respective classes, the research team then presented the rationale of the research to students of different classes. Students were instructed to seek permission from their parents to participate, and their parents’ signed consent was required on the consent form students took home. When students returned the completed consent forms the following day, they were given an assent form to ensure that their participation was completely voluntary. After obtaining proper consent and assent from parents and students respectively, data were collected from 2313 students. The data collection procedure was conducted in classrooms with the presence of a schoolteacher, lead researcher, and study team member. Students who did not take part in the study did activities of their choice during data collection.

### Outcome measure

Stress symptoms were measured using the 10-item Perceived Stress Scale (PSS-10). The PSS-10measures the degree to which one appraises situations in life as stressful [10]. The PSS-10 has been shown to be an acceptable self-reported tool for screening stress in adolescents within community settings [10–12]. Items ask how often respondents have experienced each of 10 symptoms during the past month (e.g., felt upset, felt nervous and stressed, felt unable to control important things in life, felt confident, etc.) using a 5-point Likert scale, with 0 indicating ‘never,’ 1 = almost never, 2 = sometimes, 3 = fairly often, and 4 indicating ‘very often.’ A total score for each adolescent was obtained by summing the scores across all 10 items for a total range of 0 to 40. A higher score indicated higher perceived stress, with a score of 14–26 used to indicate the presence of moderate stress and 27 or more indicating high stress [13].

Standardized Bangla version of PSS-10, achieved by translation from original English version, was utilized in this study. Following the ‘state of the art procedures’ of translation, the standardized Bangla version of the questionnaire was prepared [14]. In keeping with the study objective, the questionnaire was repeatedly revised and reviewed by the research team. Additionally, for the finalization of the questionnaire, an expert questionnaire review panel comprising a professor of public health, a professor of statistics, and two other experts in public health research was formed, who reviewed and edited the questionnaire so that it meets the study objectives. Based on the feedback of the review panel, the questionnaire was modified and sent back to them for the final draft for pre testing.

### Other measures

#### Sociodemographic variables

Data on gender, age, grade in school, birth order, parent education, family size, and residential setting were collected. During data collection, it was discovered that many students did not know their family’s socioeconomic status, so the researchers excluded these questions from the survey.

#### Lifestyle factors

Following a modified Leisure Time Exercise Questionnaire (LTEQ) and the WHO Global Physical Activity Questionnaire (GPAQ) physical activity and screen based behaviors were measured in this study [15, 16]. Physical activity (PA), Screen-Based Sedentary Behaviour (SBSB), and sleep patterns were measured to assess the lifestyle of the adolescents. Three different PA levels were in the questionnaire: low-level PA (unintentional walking < 30 min/day), moderate PA (walking or meditation/yoga ≥30 min/day), and vigorous PA (jogging, cycling, playing sports, or gym workouts ≥60 min/day) [17–19]. To measure SBSB, adolescents were asked how many hours they spent on various medias, such as Facebook, Twitter, Instagram, YouTube, or watching television. High screen time was regarded as > 2 hours/day, which is in line with commonly used screen time guidelines [20].

### Statistical analysis

To achieve the objectives of the study both descriptive and inferential statistical analyses were done. To find out the prevalence of stress symptoms among the adolescents, descriptive analysis, such as, frequency, mean etc. were done. After that, to identify any association between different risk factors, inferential analyses, e.g., chi square and logistic regression were conducted. The chi-square test was used to identify any significant relationship between the study variables. Logistic regression models were used to detect any significant associations of the study variables with the outcome variable. Necessary assumptions of regression: independence of errors, linearity in the logit for continuous variables, absence of multicollinearity, and lack of strongly influential outliers, were checked prior to the logistic regression [21]. For the final regression model, adjusted odds ratios (AORs) with 95% Confidence Intervals (CIs) were reported after adjusting for various factors. The level of significance was set at 0.05. The PSS-10 scores were turned into a binary factor, with a cut-point of ≥14 used to differentiate between those who were stressed vs. non-stressed. The Statistical Package for the Social Sciences (SPSS) software for Windows, version 26.0 (IBM, Armonk, NY, USA) was used to analyse all data.

## Results

### Prevalence of stress symptom among adolescents

**Fig. 3:**
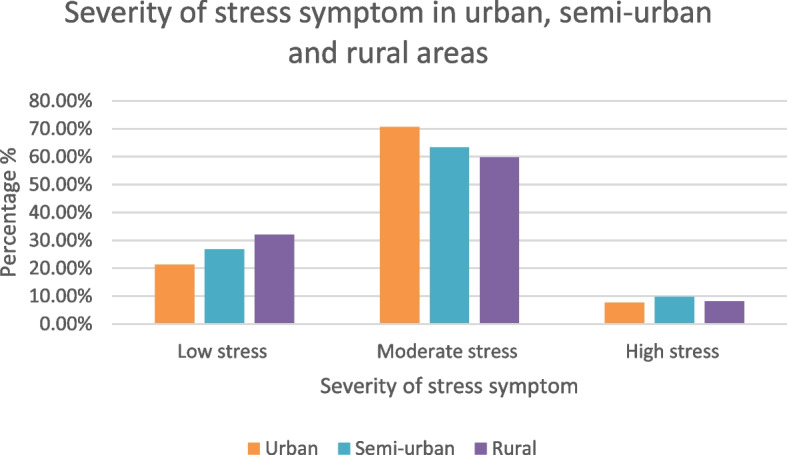
Severity of stress symptom in urban, semi-urban and rural areas of Dhaka, Bangladesh

The overall prevalence of stress symptoms among adolescents was found to be 73.5% (considering PSS total score 0–13 as stress negative and ≥ 14 as stress positive). Figure 3 demonstrates the severity of stress symptoms among urban, semi-urban and rural adolescents in Dhaka, Bangladesh. Low stress was found to be prevailing more among rural adolescents (32.00%). On the other hand, semi-urban adolescents were the highest in number suffering from high stress, with 10% of them were found to be experiencing that. Lastly, moderate stress was more prevalent among urban adolescents (70.70%) than among semi-urban and rural adolescents.

### Sociodemographic characteristics of adolescents

In this study, about 40% of the adolescents were from the urban area, while almost 32% were from semi-urban and rural areas respectively [22]. Approximately 52% of the participants were female and the rest of them were male. More than a third (32.2%) of the respondents were of 14 years of age and about two fifths (42.5%) of the participants were studying in class 9. Besides, a larger portion (51.1%) of the respondents reported to be the first child of their parents [22]. In case of parental characteristics, about 40% of the fathers completed graduation or above, while almost 30% of the mothers completed secondary/higher secondary level of education. The fathers and mothers were mostly service holders (43.3%) and homemaker (81.5%) respectively. More than three fifths (62%) of the adolescents reported that they had 4 members or less than that in their families [22].

### Relationship between sociodemographic variables and stress symptoms

Table 1 represents associations between sociodemographic variables and stress symptoms. Female gender was found to be significantly associated (χ^2^: 131.47; *p*: < 0.001) with stress symptoms, since more than half of them (58.7%) were found to be experiencing the symptoms. Likewise, age (χ^2^: 24.42; *p*: < 0.001) and student’s grade (χ^2^: 10.68; p: 0.005) were also found to have a statistically significant link with stress symptoms. Besides, residential setting was also found to have significant association (χ^2^: 22.50; *p*: < 0.001) with stress symptoms, and more than 30% of the urban and semi-urban adolescents were reported to be stressed.

**Table 1:** Relationship between sociodemographic variables and stress symptoms

Variables	Stress symptom^**a**^	
	Frequency (% within column)	χ^**2**^ value (***p***-value)
**Gender**		
Male	701 (41.3)	131.47(< 0.001)^**^
Female	998 (58.7)	
**Age in years**		
12	46 (2.7)	24.42 (< 0.001)^**^
13	256 (15.1)	
14	539 (31.7)	
15	539 (31.7)	
16	276 (16.2)	
17	43 (2.5)	
**Student grade**		
Class 8	575 (33.8)	10.68 (0.005)^**^
Class 9	733 (43.1)	
Class 10	391 (23.0)	
**Birth order**		
1st	859 (50.6)	1.06 (0.589)
2nd	579 (34.1)	
≥ 3rd	261 (15.4)	
**Father’s level of education (*n*** **= 1450)**		
Primary education	121 (11.1)	4.69 (0.096)
Secondary/ Higher secondary education	372 (34.2)	
Graduation/above	594 (54.6)	
**Mother’s level of education (*n*** **= 1486)**		
Primary education	193 (17.2)	1.88 (0.391)
Secondary/ Higher secondary education	505 (44.9)	
Graduation/above	426 (37.9)	
**Total number of family members**		
≤ 4 members	1038 (61.1)	2.01 (0.157)
≥ 5 members	661 (38.9)	
**Residence**		
Urban	652 (38.4)	22.50 (< 0.001)^**^
Semi-urban	544 (32.0)	
Rural	503 (29.6)	

^a^A cut-off of ≥14 for the PSS-10 is used for analysis

^**^p-value is significant at *p* < 0.01

### Relationship between lifestyle variables and stress symptoms

Table 2 illustrates associations between the lifestyle factors of the students and stress symptoms. Concerning physical activity (PA), more than 70% of adolescents who were not involved in PA (χ^2^: 7.12; *p*: 0.008), did irregular PA (χ^2^: 4.84; *p*: 0.028) and reported doing PA < 30 min/day (χ^2^:28.96; *p*: < 0.001), were found to have statistically significant association with stress symptoms. Similarly, a statistically significant association between stress symptoms and screen-based recreation (χ^2^: 7.77; p: 0.005) as well as sleep dissatisfaction (χ^2^: 14.39; *p*: < 0.001) was found among approximately three quarters of the adolescents.

**Table 2:** Relationship between lifestyle variables and stress symptoms

Variables	Stress symptom^**a**^	
	Frequency (% within variable)	χ^**2**^ value (***p***- value)
**Physical activity (PA)**		
**Involved in PA**		
Yes	1054 (71.6)	7.12 (0.008)^**^
No	645 (76.7)	
**Regular PA**		
Yes	1024 (71.9)	4.84 (0.028)^*^
No	675 (76.0)	
**Duration of daily PA (*n*** **= 1472)**		
< 30 min/day	1108 (76.8)	28.96 (< 0.001)^**^
30–60 min/day	375 (71.2)	
> 60 min/day	216 (63.0)	
**PA time (*n*** **= 1472)**		
Early morning of the day	346 (73.2)	14.77 (0.002)^**^
Late afternoon of the day	128 (81.0)	
Evening of the day	550 (69.3)	
**Screen based sedentary behaviour (SBSB)**		
**Use of social media (e.g., Facebook)**		
Yes	1054 (74.0)	0.50 (0.481)
No	645 (72.6)	
**Screen based recreation (Movies, video games etc.)**		
Yes	1575 (74.2)	7.77 (0.005)^**^
No	124 (64.9)	
**Duration of daily SBSB**		
≤ 2 hours/day	946 (71.9)	3.86 (0.049)^*^
> 2 hours/day	753 (75.5)	
**Sleep patterns**		
**Sleep satisfaction**		
Yes	1082 (71.0)	14.39 (< 0.001)^**^
No	617 (78.3)	
**Sleep duration**		
Short sleep duration (< 7 hours/day)	691 (76.0)	6.18 (0.045)^*^
Ideal sleep duration (7–9 hours/day)	900 (71.4)	
Long sleep duration (> 9 hours/day)	108 (75.5)	

^a^A cut-off of ≥14 for the PSS-10 is used for analysis

^**^p value is significant at *p* < 0.01

^*^p value is significant at *p* = 0.05

### Association between study variables and stress symptoms

Table 3 details associations between stress symptoms and study variables from the logistic regression analyses. Both bivariate and multivariate analyses were conducted. Variables were first adjusted for all presented sociodemographic variables (age, gender, grade, residence), and then adjusted for PA, SBSB, sleep patterns, and body perception variables. After this final adjustment for sociodemographic and lifestyle variables, the odds ratios changed slightly.

**Table 3:** Bivariate and multivariate regressions for association between study variables and stress symptoms among school adolescents in Dhaka, Bangladesh^a^

Variables	Unadjusted estimates			Adjusted estimates-1^**b**^			Adjusted estimates-2^**c**^		
	Odds ratio	95% CI	***p***-value	Odds ratio	95% CI	***p***-value	Odds ratio	95% CI	***p***-value
**Socio-demographic variables**									
**Gender**									
Female	3.06	2.52–3.72	< 0.001	3.35	2.74–4.09	< 0.001	3.13	2.53–3.89	< 0.001
Male	1.00			1.00			1.00		
**Student grade**									
Class 10	1.49	1.04–1.57	0.002	1.17	0.83–1.65	0.382	1.41	1.08–1.82	0.011
Class 09	1.28	1.15–1.97	0.021	1.23	0.86–1.57	0.101	1.29	1.04–1.59	0.018
Class 08	1.00			1.00			1.00		
**Age**									
≥ 15 years	1.36	1.13–1.64	0.001	1.38	1.06–1.78	0.015	1.30	1.08–1.58	< 0.001
< 15 years	1.00			1.00			1.00		
**Residence**									
Urban	1.73	1.38–2.17	< 0.001	1.94	1.53–2.47	< 0.001	1.53	1.20–1.95	0.001
Semi-urban	1.29	1.03–1.61	0.027	1.35	1.06–1.72	0.013	1.18	0.93–1.48	0.175
Rural	1.00			1.00			1.00		
**Lifestyle variables**									
**Engaged in PA**									
No	1.31	1.07–1.59	0.008	1.01	0.81–1.24	0.957	1.01	0.79–1.30	0.926
Yes	1.00			1.00			1.00		
**Regular PA**									
No	1.24	1.02–1.50	0.028	0.96	0.78–1.18	0.712	0.91	0.71–1.18	0.482
Yes	1.00			1.00			1.00		
**Level of PA**									
Inactive/low levels of PA	1.56	1.29–1.88	< 0.001	1.06	0.86–1.30	0.581	1.52	1.26–1.84	< 0.001
Moderate/vigorous levels of PA	1.00			1.00			1.00		
**Use of social media (e.g., Facebook)**									
Yes	1.07	0.89–1.29	0.481	1.22	0.99–1.50	0.058	1.01	0.81–1.26	0.944
No	1.00			1.00			1.00		
**Duration of daily SBSB**									
> 2 hours/day	1.21	1.00–1.46	0.050	1.26	1.03–1.55	0.028	1.18	0.97–1.42	0.097
≤ 2 hours/day	1.00			1.00			1.00		
**Sleep satisfaction**									
No	1.48	1.21–1.81	< 0.001	1.19	0.96–1.48	0.115	1.33	1.07–1.65	0.009
Yes	1.00			1.00			1.00		
**Sleep duration**									
Short sleep duration (< 7 hours/day)	1.27	1.05–1.55	0.016	1.09	0.89–1.34	0.384	1.14	0.93–1.40	0.210
Long sleep duration (> 9 hours/day)	1.24	0.83–1.85	0.296	1.27	0.84–1.92	0.268	1.15	0.77–1.73	0.492
Ideal sleep duration (7–9 hours/day)	1.00			1.00			1.00		
**Body image dissatisfaction**^**d**^									
Yes	1.41	1.14–1.73	0.002	1.28	1.03–1.59	0.029	NA		
No	1.00			1.00					
**Perceived weight category**									
Over-weight/obese	1.59	1.23–2.05	< 0.001	1.34	1.03–1.76	0.031	1.46	1.13–1.89	0.004
Underweight	1.14	0.83–1.58	0.423	1.12	0.80–1.57	0.519	1.03	0.74–1.43	0.847
Normal	1.00			1.00			1.00		

*NA* Not applicable (excluded from the multivariate model since their P value was greater than 0.1

^a^Estimates are based on a binary logistic regression with stress symptoms (≥14 scores on PSS-10) as the dependent variable

^b^Adjusted for all presented socio-demographic variables (age, gender, grade, residence) in the table

^c^Adjusted for level of PA, duration of daily screen time, satisfaction with daily sleep, sleep habits, and perceived weight category

^d^Body image dissatisfaction was highly correlated with perceived weight category (*r* = −0.88) and removed from the multivariate model

According to adjusted estimate-1, female adolescents had three times greater risk (AOR: 3.35; 95% CI: 2.74–4.09) of experiencing stress symptoms compared to their male counterparts. Besides, older adolescents (≥15 years) were at 1.38 times (95% CI: 1.06–1.78) higher risk of suffering from stress symptoms than adolescents of < 15 years. In addition, urban adolescents were about two times (AOR: 1.94; 95% CI: 1.53–2.47) more likely to suffer from stress symptoms in compared to rural adolescents, while adolescents from semi-urban area were at 1.35 times higher risk (95% CI: 1.06–1.72) of experiencing stress symptoms than rural adolescents.

In adjusted estimate-2, it has been demonstrated that inactive adolescents or adolescents who were doing low-level PA were at 1.52 times (95% CI: 1.26–1.84) more risk of experiencing stress symptoms than adolescents who reported doing moderate to vigorous PA. Again, adolescents dissatisfied with their sleep were at higher risk (AOR: 1.33; 95% CI: 1.07–1.65) of suffering from stress symptoms than those satisfied with their sleep.

## Discussion

Findings from this study suggest that moderate to high stress symptoms are common among secondary school-going adolescents in Bangladesh, with a concerning prevalence estimate of 73.5%. As studies on the prevalence rates of stress symptoms among secondary school-aged adolescents in Bangladesh could hardly be found, an extensive literature search was conducted to find available estimates of similar populations. The prevalence estimate of stress symptoms among adolescents in the present study is much higher than that(24.9%) of a study conducted on 590 university students in 2019 in Bangladesh [23]. Another Bangladeshi study with 105 medical students from 2017 also reported a lower prevalence (59.0%) of stress than that of the present study [24]. University-aged youths may have more developed coping mechanisms for stress than secondary school-aged adolescents. Nonetheless, this discrepancy merits further research. Compared to neighboring countries, the prevalence of stress symptoms in the current study is lower than that (87.6%) reported in a study conducted in Delhi, India [25]. However, a study [26] on secondary school adolescents in Delhi, India reported a slightly lower prevalence of stress (60.9%) than the prevalence reported in the current study. Again, the prevalence estimate reported in this study is also 6% higher than that of a 2019 study conducted among secondary school adolescents who had internet addiction [27]. In a Saudi study of adolescents addicted to gaming, only 11% of the participants experienced stress [28]. These variations difference in the prevalence reported in the above-mentioned studies as well as in the current study might be attributable to differences in measurements, study settings, or targeted populations. With associations between screen use and stress being found insignificant in the current study, further research is needed to investigate any link between screen-use behavior and stress among adolescents.

In the present study, female adolescents had more than three times the odds of suffering from stress symptoms than their male counterparts, which is in keeping with the findings of another study [29]. This specific finding is also consistent with the updated mental health country profile of Bangladesh [30]. This may be explained by factors such as parent-adolescent relationships and inefficacy in academia affecting female adolescents more than male adolescents [31]. Emotional turmoil during this period, physiological changes, and negative gender stereotypes within society might also play important roles as stressors among female adolescents. The odds of experiencing stress significantly rose with grade level and older age among secondary school adolescents in the current study. Given the excessive academic pressures of upcoming graduation examinations in higher grades and increasing family expectations for academic success, this finding is unsurprising. This study suggests that urban adolescents have higher odds of experiencing stress than rural adolescents, which is consistent with the findings of another study [32]. Recent findings suggest that urbanicity does not impact mental health directly, but it is associated with acute autonomic nervous system and hypothalamic-pituitary-adrenal axis reactivity. Apart from this, increased social stress has been observed more in urban environments compared to rural environments, and living in urban environments has been associated with dysregulated stress system functioning [33, 34]. Findings from neuroimaging studies show that adults who grew up and currently live in urban areas demonstrate differential limbic brain area responsivity towards psychosocial stress in comparison to adults living in and brought up in rural areas, although the exact mechanism for this is still unknown [35, 36].

Those who were inactive or engaged in low levels of PA had higher odds of experiencing stress than those who were very physically active. There is an established bidirectional relationship between stress and PA. Studies show that moderate to vigorous PA is helpful in managing stress among secondary school adolescents [37]. On the other hand, stressed adolescents are less likely to engage in PA [38]. The association found between daily sleep satisfaction and stress among adolescents in this study is also consistent with the findings of other studies [39, 40]. Studies have shown school stress, technology use, and academic overload as factors contributing to sleep dissatisfaction among adolescents, which suggests that sleep may also have a bidirectional relationship with stress [39, 41]. Regression analyses from the current study suggest that self-body image plays an important role in understanding stress among secondary school adolescents. Body image dissatisfaction may trigger peer pressure and bullying [42], contributing to feelings of inferiority among adolescents. In the current study, adolescents who perceived themselves as overweight or obese had higher odds of experiencing stress than those who perceived themselves as normal, which is consistent with the literature [29]. Experiencing bullying due to one’s perceived body weight has been found to cause stress among adolescents [43].

## Strengths and limitations

Although stress is an alarming public health issue, very few studies have explored stress symptoms among adolescents in Bangladesh. Many of the existing studies cover only one type of geographical region, without comparing urban, semi-urban, and rural areas simultaneously. To the best of our knowledge, this is till now the only study found to investigate stress symptoms among secondary school adolescents from urban, semi-urban, and rural areas in Bangladesh. The diversity of areas covered makes the findings of this study useful in reformulating local policies that impact adolescent mental health. The comprehensive data collection allows this study to stand out among the limited research available.

One of the limitations of this study lie in the cross-sectional nature of this study, as no causality can be established. Measures used relied on self-reported information, which always involves risks of recall and reporting bias. Although data were collected from regions representing urban, semi-urban, and rural environments, only a small portion of each area was sampled, limiting the generalizability of the findings. Furthermore, the findings of this study might also not be fully representative of populations across all districts of the country. Future research may therefore focus on conducting large-scale longitudinal studies that will cover a nationwide representative sample.

## Conclusion

Stress symptoms are highly prevalent among secondary-school adolescents in Bangladesh. Immediate and adequate interventions are needed to address the issue of stress prevalent among adolescents. Findings suggest a need to test if interventions to encourage modification of different lifestyle factors among adolescents will improve their mental health.

## Supplementary Information

**Additional file 1.**

**Additional file 2.**

## Data Availability

The datasets used and/or analyzed during the current study are available from the corresponding author on reasonable request.
