# Supplementary material for: Stress symptoms and associated factors among adolescents in Dhaka, Bangladesh: findings from a cross-sectional study
Source: BMC Psychiatry. 2022 Dec 19;22:807. doi: 10.1186/s12888-022-04340-0 (PMC9761971; doi:10.1186/s12888-022-04340-0)
Supplement: Supplementary file 1 — Additional file 1. [file 12888_2022_4340_MOESM1_ESM.pdf]

Investigating Prevalence of and Factors Associated with Stress Symptoms among Urban, Semi-urban and Rural School Adolescents in Dhaka District of Bangladesh

Section A: Socio-Demographic Information

Serial no.	Questions	Answer/Coding categories
A_1	What is your class roll number?	
A_2	What is your class/grade?	
A_3	Mention your age in years	
A_4	What is your gender?	1=Male<br>2=Female<br>3=Others
A_5	What is your birth order?	1= First child<br>2= Second child<br>3= Third child<br>4= Others (Please specify)<br>--------------------------------
A_6	What is your religion?	1= Islam<br>2= Hinduism<br>3= Christianity<br>4= Buddhism
A_7	What is your father's occupation?	
A_8	What is father's educational qualification?	
A_9	What is your mother's occupation?	
A_10	What is your mother's educational qualification?	
A_11	Please mention the number of your family members	
A_9	Please mention your family's monthly income in taka	

Section B: Quality of Health (Self-reported health/body image)

Serial no.	Questions	Answer/coding categories
B_1	In general, would you say your health is---	1= Excellent<br>2= Very good<br>3= Good<br>4= Fair<br>5= Poor
B_2	Are you satisfied with your current sleeping manner?	1= Yes<br>2= No
B_3	At present, how many hours do you sleep?	---------------- Hours

B_4	Do you have any dissatisfaction with your body weight?	1=Yes<br>2=No
B_5	Body image:<br>How you perceive your current body weight?	1= Underweight<br>2= Normal/Healthy Weight<br>3= Overweight<br>4= Obese

Section C: Physical Activity

C_1	Do you have practice to do any physical activity?	1=Yes<br>2=No
C_2	If yes, please mention what type of activity you are doing?	1= Walking<br>2= Jogging (including, dancing and skipping)<br>3= Cycling<br>4= Playing sports (e.g., cricket, football, basketball, handball, badminton and table tennis)<br>5= Gym workouts (including, Karate)<br>6= Meditation/yoga<br>7=Others (Pl. Specify)
C_3	If yes, do you do PA regularly?	1=Yes,<br>2=No<br>3= Sometimes
C_4	Over the last 7 days, how often did you involved in PA? (mention days)	
C_5	On average, how long do you spend in PA in a day?	1= less than 30 min.<br>2= 30min to 1 hour.<br>3= 1 to 2 hours.<br>4= More than 2 hours
C_6	By which time of the day would you like to do PA?	1= Early morning of the day (between 5 to 8 am)<br>2= Late afternoon of the day (between 4 to 6 pm)<br>3= Evening of the day (between 6 to 9 pm)

Section D: Screen Based Sedentary Behavior

D_1	Which kind of cell phone are you using?	1= Android<br>2= iPhone<br>3= Normal Phone<br>4= Don’t use any phone		
D_2	Are you using any type of social media (like Facebook, Twitter, Instagram etc.)?	1=Yes<br>2=No		
D_3	On average how long do you spend social media?	(a) Daily (h)	(b) Weekly (h)	(c) Monthly (h)
D_4	Do you watch movie/play/live or YouTube video game for recreation?	1=Yes<br>2=No		
D_5	If yes, mention when and how long?	(a) Daily (h)	(b) Weekly (h)	
D_6	What is the main method by which you use Facebook and watch movies/plays?	1= Android mobile phone;<br>2= Computer<br>3= Television		

Section E: Psychological Health

Instruction: For the last 30 days, how much you have felt towards the following statements. Please put a tick mark (✓) in the respective columns.

**Perceived Stress Scale**						
Serial no.	In the last month	Never (0)	Almost never (1)	Sometimes (2)	Fairly often (3)	Very often (4)
E_1	How often have you been upset because of something that happened unexpectedly					
E_2	How often have you felt that you were unable to control the important things in your life?					
E_3	How often have you felt nervous and “stressed”?					
E_4	How often have you felt confident about your ability to handle your personal problems?					
E_5	How often have you felt that things were going your way?					
E_6	How often have you found that you could not cope with all the things that you had to do?					

E_7	How often have you been able to control irritations in your life?					
E_8	How often have you felt that you were on top of things?					
E_9	How often have you been angered because of things that were outside of your control?					
E_10	How often have you felt difficulties were piling up so high that you could not overcome them?					
