# Supplementary material for: Stress symptoms and associated factors among adolescents in Dhaka, Bangladesh: findings from a cross-sectional study
Source: BMC Psychiatry. 2022 Dec 19;22:807. doi: 10.1186/s12888-022-04340-0 (PMC9761971; doi:10.1186/s12888-022-04340-0)
Supplement: Supplementary file 2 — Additional file 2. [file 12888_2022_4340_MOESM2_ESM.pdf]

Sample size determination estimation

$$n = \frac{z^2\, pq}{d^2} \times 2$$

$$n = \frac{(1.96)^2 \times 0.366 \times (1 - 0.366)}{(0.05)^2} \times 2$$

$$n = 713.14$$

$$n \approx 713$$

n= Total sample size
z= 1.96 (for 95% Confidence Interval)
p= 0.366 (previous mental disorder rate among school adolescents)[10]
q= (1-p), = (1-0.366), = 0.634
d= 0.05 (where 5% margin of error was accepted)
DE= Cluster design effect = 2

Reference:

10. Anjum A, Hossain S, Sikder T, Uddin ME, Rahim DA. Investigating the prevalence of and factors associated with depressive symptoms among urban and semi-urban school adolescents in Bangladesh: a pilot study. Int Health. 2019. Epub 20191106. doi: 10.1093/inthealth/ihz092. PubMed PMID: 31693088
